# Exosomes Derived From Hypoxia-Conditioned Stem Cells of Human Deciduous Exfoliated Teeth Enhance Angiogenesis *via* the Transfer of let-7f-5p and miR-210-3p

**DOI:** 10.3389/fcell.2022.879877

**Published:** 2022-04-26

**Authors:** Panpan Liu, Lihong Qin, Chang Liu, Jun Mi, Qun Zhang, Shuangshuang Wang, Dexuan Zhuang, Qiuping Xu, Wenqian Chen, Jing Guo, Xunwei Wu

**Affiliations:** ^1^ Department of Tissue Engineering and Regeneration, School and Hospital of Stomatology, Cheeloo College of Medicine, Shandong University & Shandong Key Laboratory of Oral Tissue Regeneration & Shandong Engineering Laboratory for Dental Materials and Oral Tissue Regeneration, Jinan, China; ^2^ Department of Pediatrics Dentistry, Department of Preventive Dentistry, School and Hospital of Stomatology, Cheeloo College of Medicine, Shandong University & Shandong Key Laboratory of Oral Tissue Regeneration & Shandong Engineering Laboratory for Dental Materials and Oral Tissue Regeneration, Jinan, China; ^3^ Department of Stomatology, Weihai Hospital of Traditional Chinese Medicine, Weihai, China; ^4^ Department of Orthodontics, School and Hospital of Stomatology, Cheeloo College of Medicine, Shandong University & Shandong Key Laboratory of Oral Tissue Regeneration & Shandong Engineering Laboratory for Dental Materials and Oral Tissue Regeneration, Jinan, China; ^5^ Engineering Laboratory for Biomaterials and Tissue Regeneration, Ningbo Stomatology Hospital, Ningbo, China; ^6^ Savaid Stomatology School, Hangzhou Medical College, Hangzhou, China

**Keywords:** exosome, stem cells of human deciduous exfoliated teeth, hypoxia, angiogenesis, let-7f-5p, miR-210-3p

## Abstract

Physiological root resorption of deciduous teeth is a normal phenomenon. How the angiogenesis process is regulated to provide adequate levels of oxygen and nutrients in hypoxic conditions when the dental pulp tissue is reduced at the stage of root resorption is not fully understood. In this study, we designed hypoxic preconditioning (2%) to mimic the physiological conditions. We isolated exosomes from hypoxic-preconditioned SHED (Hypo-exos) cells and from normally cultured SHED cells (Norm-exos). We found that treatment with Hypo-exos significantly enhanced the growth, migration and tube formation of endothelial cells *in vitro* compared with Norm-exos. We also performed matrigel plug assays *in vivo* and higher expression of VEGF and higher number of lumenal structures that stained positive for CD31 were found in the Hypo-exos treated group. To understand the potential molecular mechanism responsible for the positive effects of Hypo-exos, we performed exosomal miRNA sequencing and validated that Hypo-exos transferred both let-7f-5p and miR-210-3p to promote the tube formation of endothelial cells. Further study revealed that those two miRNAs regulate angiogenesis *via* the let-7f-5p/AGO1/VEGF and/or miR-210-3p/ephrinA3 signal pathways. Finally, we found that the increased release of exosomes regulated by hypoxia treatment may be related to Rab27a. Taking these data together, the present study demonstrates that exosomes derived from hypoxic-preconditioned SHED cells promote angiogenesis by transferring let-7f-5p and miR-210-3p, which suggests that they can potentially be developed as a novel therapeutic approach for pro-angiogenic therapy in tissue regeneration engineering.

## Introduction

Physiological root resorption in deciduous teeth is a normal phenomenon that is necessary for the shedding of deciduous teeth and the eruption of permanent teeth (Sahara, 2001). To date, many studies have focused on inflammation and osteoclastogenesis during the stages of root resorption (Wang L et al., 2017). At the onset of physiological root resorption, the blood vessels in the dental pulp tissue are reduced and oxygen availability is decreased. The underlying mechanism of how the angiogenesis process is regulated to provide adequate levels of oxygen and nutrients in such an ischemic scene is not fully understood. It has been shown that a variety of paracrine factors are produced by mesenchymal stem cells (MSCs) cultured in hypoxic conditions to exert therapeutic repair for ischemic tissues (Pankajakshan and Agrawal, 2014).

As a major contributor to the paracrine effects of MSCs, exosomes (30–200 nm) are small extracellular bilayer membrane-bound vesicles of endocytic origin that can be discharged into the extracellular microenvironment once they fuse with the plasma membrane (Harding et al., 1983; van Niel et al., 2006). Exosomes are produced by all types of cells, and they transport proteins, mRNAs and miRNAs to recipient cells to modulate their biological activities, such as the angiogenesis process (Balaj et al., 2011; Webber et al., 2015; Qin et al., 2016).

Oxygen has been shown to play critical roles in the survival of embryonic and adult stem cells. The cellular microenvironment is regulated by different concentrations of oxygen, which can serve as a signaling molecule and as a metabolic substrate (Abdollahi et al., 2011). Traditionally, the oxygen tension of standard laboratory culture conditions is 21% *in vitro*, which is much higher than the physiological oxygen microenvironment for cells *in vivo*. It has been reported that the relative oxygen tension of arterial blood is around 12% and varies from about 3% to 7.4% depending on the various locations of normal tissues (McKeown, 2014). The oxygen tension of the lower incisor pulp in rats was found to approximately 3% (Yu et al., 2002). Hypoxia preconditioning can activate hypoxia-inducible factor (HIF-1a), which modulates the expression of angiogenic factors, such as vascular endothelial growth factor (VEGF), a powerful regulator in angiogenesis (Majmundar et al., 2010).

As the microenvironment where MSCs are cultured can considerably influence the molecular cargoes of exosomes (Hofmann et al., 2012), hypoxic cultures of MSCs can be strategically applied to alter the exosomal contents to regulate angiogenesis. Han et al. demonstrated that exosomes obtained from hypoxia-treated human adipose tissue-derived MSCs enhanced angiogenesis in fat grafts, mainly *via* the VEGF/VEGFR signaling pathway (Han et al., 2019). Liu et al. found that hypoxic (5% O_2_) human umbilical cord MSCs-derived exosomes promote angiogenesis and improve the healing of bone fractures by the transfer of miR-126 through the activation of HIF-1a (Liu et al., 2020a). Another study revealed that exosomes from hypoxia treated (0.5% O_2_) bone MSCs augment neovascularization and myocardial reparative functions by conveying miRNA-210 (Zhu et al., 2018). Specifically, the effects of hypoxic preconditioning on the different types of MSCs are diverse and the underlying mechanism on angiogenesis remains to be clarified.

Thus, we used hypoxic culture conditions (2% O_2_) *in vitro* to mimic the physiological cellular microenvironment *in vivo* at stages of the shedding of deciduous teeth. We hypothesized that exosomes from hypoxic-preconditioned SHED cells would enhance angiogenesis to compensate for the physiologically ischemic conditions when physiological root resorption and blood vessel lessening occur. Therefore, the aim of our study was to explore how the hypoxic treatment of SHED cells modulates exosomal contents to affect angiogenesis.

## Materials and Methods

### Cell Isolation and Cultivation

Stem cells of human deciduous exfoliated teeth were isolated from eight donors with discarded deciduous incisors with at least more than one-third of the physiological root. SHED cells were cultured in Dulbecco’s Modified Eagle medium (DMEM, Cat. 11995500, Thermo Fisher Scientific) containing 1% penicillin-streptomycin (Cat. 10378016, Thermo Fisher Scientific) and 10% fetal bovine serum (FBS, Cat. 16140071, Thermo Fisher Scientific) as previously described (Werle et al., 2016). Written informed consent was given by the guardians of the donors. The procedure for obtaining discarded deciduous incisors was approved by the Medical Ethical Committee of the School of Stomatology Shandong University (No. 20190919, Date: 02-23-2021). SHED cells at passages three to six were used for all experiments. The normoxic conditions were designed as 37°C under 5% CO_2,_ 21% O_2_ and the hypoxic conditions of 2% O_2_ were controlled by a C-chamber incubator (ProOx P110 O_2_ Controllers, BioSperix). Human umbilical vein endothelial cells (HUVECs) were purchased from AllCells (Cat. H-001F, AllCells) and were cultured in DMEM (Cat. SH30021.01, Hyclone) supplemented with 10% FBS and 1% penicillin-streptomycin. HUVECs were cultured at 37°C under 5% CO_2_.

### Isolation and Characterization of Exosomes

Collected cell conditioned media were centrifuged at 3,000 × g for 10 min at 4°C followed by filtration through 0.2 μm filters as previously reported (Fujio et al., 2017). Hypoxic exosomes (Hypo-exos) and normoxic exosomes (Norm-exos) from culture supernatants were isolated and purified using an exosome concentration solution (ECS) kit (Cat. UR52121, Umibio) following the manufacturer’s protocol. Briefly, the prepared supernatants were added to ECS and stored for 2 h at 4°C after mixing. Following centrifugation at 10,000 × g for 60 min at 4°C, the precipitates were resuspended and purified using a purification filter centrifuged at 3,000 × g for 10 min at 4°C and then stored at −80°C.

A bicinchoninic acid (BCA) protein quantitation kit (Cat. PC0020, Solarbio) was used to analyze the exosomal concentration after which the exosomes were characterized. First, a transmission electron microscope (TEM, G2 spititi FEI, Tecnai) was used to observe the morphology of exosomes. Next, the particle size distribution of exosomes was analyzed *via* nanoparticle tracking analysis (NTA) using ZetaView Particle Metrix (ZetaView PMX 110, Particle Metrix). The exosome-specific markers CD63 (Cat. Ab216130, Abcam) and TSG101 (Cat. Ab125011, Abcam) were detected by western blotting.

### Uptake of Exosomes by Human Umbilical Vein Endothelial Cells

Hypo-exos and Norm-exos were labeled with PHK67 fluorescent dye (Cat. UR21028, Umibio) following the manufacturer’s instructions. In brief, 5 μl PHK67 dye was added to PBS containing 30 μg exosomes containing 50 μl diluent C and was incubated at room temperature for 10 min. After re-isolating and re-purifying, the labeled exosomes were added to HUVECs and incubated at 37°C for 24 h. An inverted fluorescence microscope (Olympus, Japan) was used to capture images of ingested exosomes.

### Cell Counting Kit-8 Assays

CCK8 assays were performed using a Cell Counting Kit-8 (Cat. 35532286, Dojindo) to assess cell viability. Briefly, HUVECs at a density of 5 × 10^3^ cells per well in 100 μl medium were seeded into 96-well plates and incubated with Hypo-exos or Norm-exos (30 μg/ml). Ten μl CCK8 solution in fresh medium was added into each well every 24 h. After incubation for 1.5 h at 37°C, the optical absorbance was quantified at 450 nm wavelength using a microplate reader (ELx800, Bio-Tek).

### Ki67 Immunofluorescent Staining

The cell proliferation ability was determined using immunofluorescence staining for Ki67. Briefly, HUVECs were seeded on a microscope cover glass in 24-well plates at a density of 2 × 10^4^ cells per well and were cultured for 24 h until they became approximately 60% confluent. After fixation with 4% paraformaldehyde for 15 min, cells were permeabilized using 0.5% Triton X-100 (Cat. 9036-19-5, Sigma-Aldrich). Then 10% goat serum was added for blocking after washing three times with PBS. Next, cells were incubated with the Ki67 primary antibody (Cat. Ab15580, Abcam) overnight at 4°C. On the second day, the cells were incubated with secondary antibodies in the dark at room temperature for at 1 h. DAPI (Cat. Ab104139, Abcam) was then used to stain the cell nuclei for 5 min. The images were captured using a BX53-DP80 immunofluorescence microscope (Olympus, Japan). The proliferation rate was calculated as follows: the number of proliferating cells/total number of cells × 100%.

### Motility and Migration Assays

The effects of Hypo-exos and Norm-exos on cell motility were determined using scratch wound healing assays. Briefly, 3 × 10^4^ HUVECs were seeded in 6-well plates. After growing to 100% confluence, cells were vertically scratched using a 200 μl pipette tip. After washing three times with PBS, fresh cell culture medium containing Hypo-exos or Norm-exos at a concentration of 30 μg/ml were added. At 0 and 24 h after scratching, the images were captured using an optical microscope (Leica, Germany).

Transwell migration assays were also used to assess the effects of Hypo-exos and Norm-exos on the migratory activity of HUVECs. In brief, 1 × 10^4^ HUVECs were seeded into the upper chamber of 24-well transwell plates with 8 μm pore size membrane (Cat. 3422, Corning). The upper chamber contained 100 μl serum-free DMEM and 500 μl medium supplemented with 10% exosomes-deprived FBS with Hypo-exos or Norm-exos (30 μg/ml) added to the lower chamber. After co-incubation for 24 h, the cells that had migrated from the upper chamber to the lower chamber were fixed with 4% paraformaldehyde for 10 min and then were stained with 0.5% crystal violet for 7 min. After removing the cells at the upper surface of the membrane, the number of stained cells was calculated to compare the migratory activity.

### Quantitative Reverse Transcription Polymerase Chain Reaction Assay

Total RNAs were extracted from cells and from SHED-derived exosomes using the Trizon reagent (Cat. 50175111, Thermo Fisher Scientific). RNA concentrations were analyzed using a Nanodrop spectrophotometer (Thermo Fisher Scientific) and each RNA was then reverse transcribed to complementary DNA (cDNA) using a superscript III first strand kit (Bio-Rad 1708891). qRT-PCR reactions were performed with the SYBR Green qPCR Mix (Cat. 9211, Biosharp) using a LightCyclerR 96 (Roche Diagnostics) with the following parameters: 95°C for 30 s, 40 cycles at 95°C for 5 s, at 60°C for 20 s and ended with an elongation step for 15 s at 72°C. GAPDH was used as an internal control. Oligo sequences of each gene used for PCR analysis are shown in Supplementary Table S1.

For miRNA qRT-PCR reactions, each RNA was reverse transcribed to complementary DNA (cDNA) using an All-in-oneTM miRNA first-stand cDNA synthesis kit (Cat. AMRT-0020, GeneCopoeia) and reaction procedures were performed using an All-in-one™ miRNA qPCR kit (Cat. AMRP-1200, GeneCopoeia) in a total volume of 10 μl. We used cel-miR-39 as the external reference (Perge et al., 2017). The relative expression levels were determined using the 2-ΔΔCt method. All primers used for miRNA qRT-PCR were purchased from GeneCopoeia.

### Western Blot Analysis

Total proteins of cells were lysed with radioimmunoprecipitation assay (RIPA, Cat. 89900, Thermo Fisher Scientific) buffer containing 1% phosphatase inhibitor cocktail and 1% phenylmethylsulfonyl fluoride (PMSF, Cat. 36978, Thermo Fisher Scientific) for 30 min at 4°C. Protein concentrations were calculated using a BCA protein quantitation kit (Cat. PC0020, Solarbio). Equal amounts of extracted proteins were loaded and separated on 12% SDS-PAGE gels and then were transferred to polyvinylidene fluoride (PVDF) membranes (Cat. 88518, Thermo Fisher Scientific) according to standard protocols. The membranes were blocked with 5% non-fat milk powder dissolved in Tris-buffered saline containing 0.05% Tween-20 (TBST, Cat. T1081, Solarbio) for at least 1 h and then were incubated with the primary antibodies overnight at 4°C. After washing with TBST buffer three times for 10 min each, the membranes were incubated with the secondary antibodies at room temperature for 1 h. Signal detection of protein bands was visualized by enhanced chemiluminescence reagents (Cat. SW 2050, Solarbio) and observed using ECL chemiluminescence (Cat. SW 2010, Solarbio) and then analyzed using ImageJ software (National Institutes of Health).

The primary and secondary antibodies used were as follows: GAPDH (Cat. Ab181602, Abcam), VEGF (Cat. Ab1316, Abcam), MMP-9 (Cat. Ab228402, Abcam), ANGPT1 (Cat. EPR2888N, Abcam), AGO1 (Cat. Ab5070, Abcam), EphrinA3 (Cat. Ab153706 Abcam), HIF-1a (Cat. 36169, Cell Signaling Technology), nSMase 2 (Cat. A10197, ABclonal), Rab27A (Cat. EM1706-32, Huabio), HRP anti-rabbit IgG (Cat. 7074, Cell Signaling Technology) and HRP anti-mouse IgG (Cat. 7076, Cell Signaling Technology).

### Tube Formation Assays

To compare effects on the angiogenic capacity of HUVECs treated with Hypo-exos or Norm-exos, tube formation assays were performed. In brief, pre-cooled 96-well plates were covered with 50 μl dissolved Matrigel (Cat. 354230, Corning). After solidification, 3 × 10^4^ HUVECs in suspension containing Hypo-exos or Norm-exos were added into each gelled Matrigel-coated well. After 12 h, the network structures of formed tubes were observed using a phase contrast microscope (Leica) and were quantified using ImageJ software.

### *In Vivo* Angiogenesis Assays

Murine matrigel plug assays were used to examine angiogenesis ability *in vivo* as described previously (Zeng et al., 2019). One × 10^7^ HUVECs resuspended in 500 μl dissolved Matrigel (Cat. 354230, Corning) containing 100 μg Hypo-exos or Norm-exos were subcutaneously injected into the dorsal skin along the abdominal midline of four-week-old female BALB/C nude mice (Cat. 403, Charles River) to form matrigel plugs. At 10 days after the injection, the mice were sacrificed, after which the matrigel plugs were excised, fixed with formalin and embedded in paraffin. The level of angiogenesis was qualified by immunohistochemical staining for CD31 (Cat. Ab28364, Abcam) and immunofluorescence staining for VEGF (Cat. Ab1316, Abcam), specific markers of micro-vessels. ImageJ software was used to quantify the VEGF expression according to the pixel.

### Sequencing Analysis of Exosomal miRNAs

To further explore the miRNA expression profiles of Hypo-exos and Norm-exos, exosomal miRNA sequencings were analyzed in three independent experiments by LC-Bio Technology. The significantly upregulated miRNAs with a ≥2 fold difference between Hypo-exos and Norm-exos were focused on.

### Transfection of miRNA Mimics, Inhibitor or Negative Control Into Human Umbilical Vein Endothelial Cells

The targeted miRNA mimics, inhibitor and negative control (NC) were transfected into HUVECs using Lipofectamine 3000 (Cat. L000001, Thermo Fisher Scientific). In brief, we mixed 12.5 μl miRNA mimics, inhibitor or negative control with 125 μl Opti-MEM™ medium (Cat. 31985088, Thermo Fisher Scientific) and incubated them for 5 min. Simultaneously, 7.5 μl Lipofectamine 3000 was diluted in 125 μl Opti-MEM™ medium and incubated for 5 min. After mixing and co-incubation for 10 min, all reagents were added to HUVECs grown in six-well plates at approximately 60% confluency. After co-incubation for 24 h, the fresh cell medium was changed. The tube formation assays were performed, and genes related to angiogenesis were determined after another 24 h.

### Statistical Analysis

Data is presented as means ± standard deviation (SD). Comparisons between Hypo-exos and Norm-exos were analyzed by Student’s t test using SPSS software (SPSS, IBM Corp. Version 22.0). A *p* < 0.05 is considered significant.

## Results

### Characterization and Internalization of Exosomes Derived From SHED Cells Cultured Under Hypoxic or Normoxic Conditions

**FIGURE 1:**
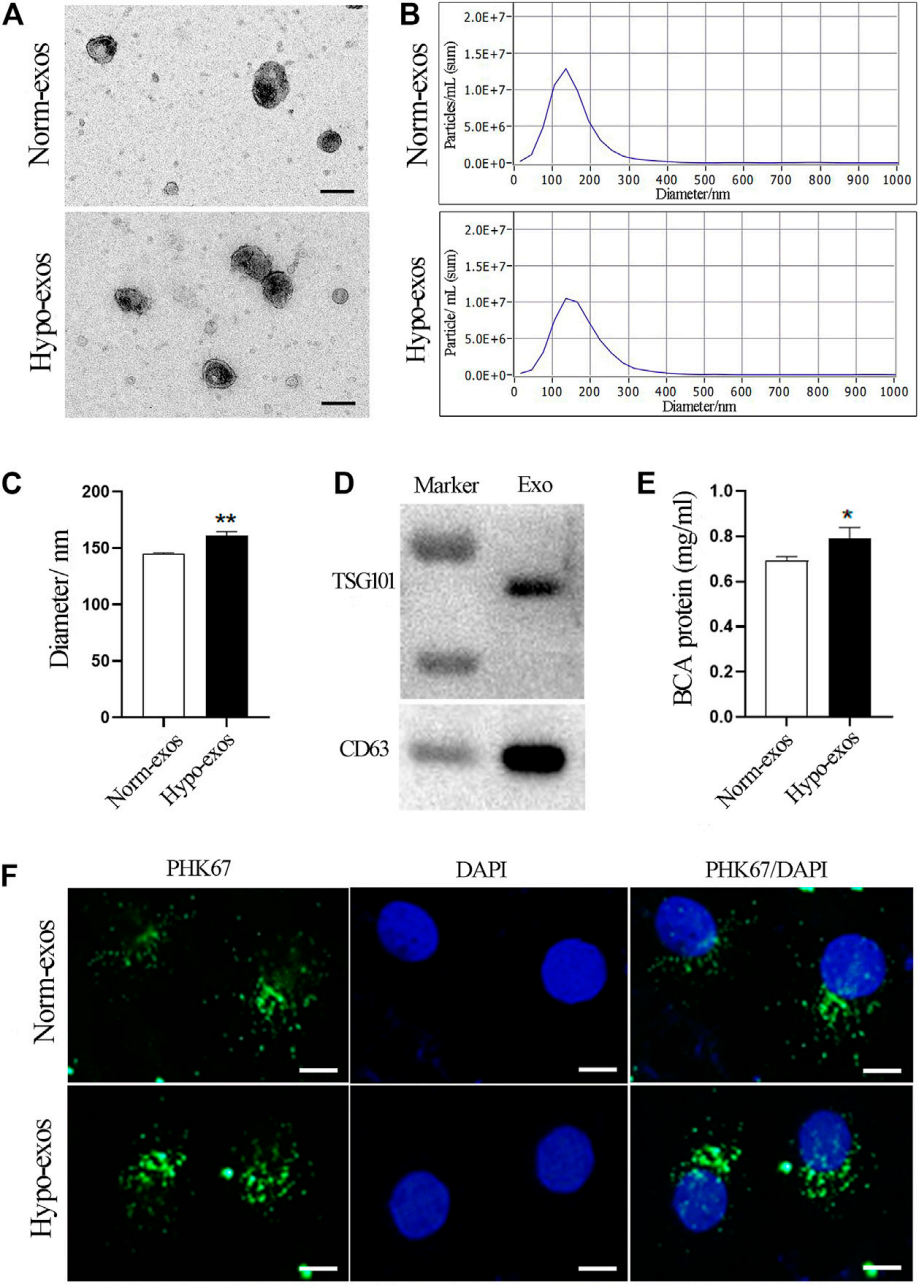
Characterization and internalization of Hypo-exos and Norm-exos. **(A)** Morphology of Hypo-exos and Norm-exos observed using transmission electron microscopy; scale bars = 100 nm. **(B)** Particle size distributions of Hypo-exos and Norm-exos were determined by nanoparticle tracking analysis. **(C)** Comparison of mean diameters of Hypo-exos and Norm-exos. **(D)** Expression of CD63 and TSG101 in exosomes validated using western blotting. **(E)** Exosomal protein concentrations in Hypo-exos and Norm-exos analyzed using the BCA assay. **(F)** Uptake of PKH67-labeled Hypo-exos and Norm-exos into HUVECs; scale bars = 200 μm *p < 0.05, **p < 0.01.

Initially, we isolated SHED cells and characterized them by analyzing specific MSC surface markers using flow cytometry (Supplementary Figure S1). Next, we characterized exosomes secreted from SHED cells under hypoxic and normoxic conditions. A similar morphology with a typical cup shape was observed in both groups by transmission electron microscopy (TEM) (Figure 1A). Nanoparticle tracking analysis (NTA) showed that the mean diameter of Norm-exos was 144.9 ± 0.80 nm while Hypo-exos were significantly larger with an average diameter of 161.0 ± 3.50 nm (Figures 1B,C). The expression of the exosomal markers CD63 and TSG101 in exosomes was detected using western blot analysis (Figure 1D). Further, we found that the exposure of SHED cells to hypoxia significantly increased the concentration of exosomes in the supernatant compared with SHED cells cultured in normoxic conditions (Figure 1E). Subsequently, to explore the potential biological functions of Norm-exos and Hypo-exos, we added those exosomes labeled with the PHK67 fluorescent dye to HUVECs. As revealed in Figure 1F, both types of PHK67-labeled exosomes were efficiently taken up by HUVECs after exposure for 24 h, indicating that the internalized exosomes could potentially produce effects on those cells.

### Hypo-Exos Significantly Enhance the Growth and Migration of Endothelial Cells *In Vitro* Compared With Norm-Exos

**FIGURE 2:**
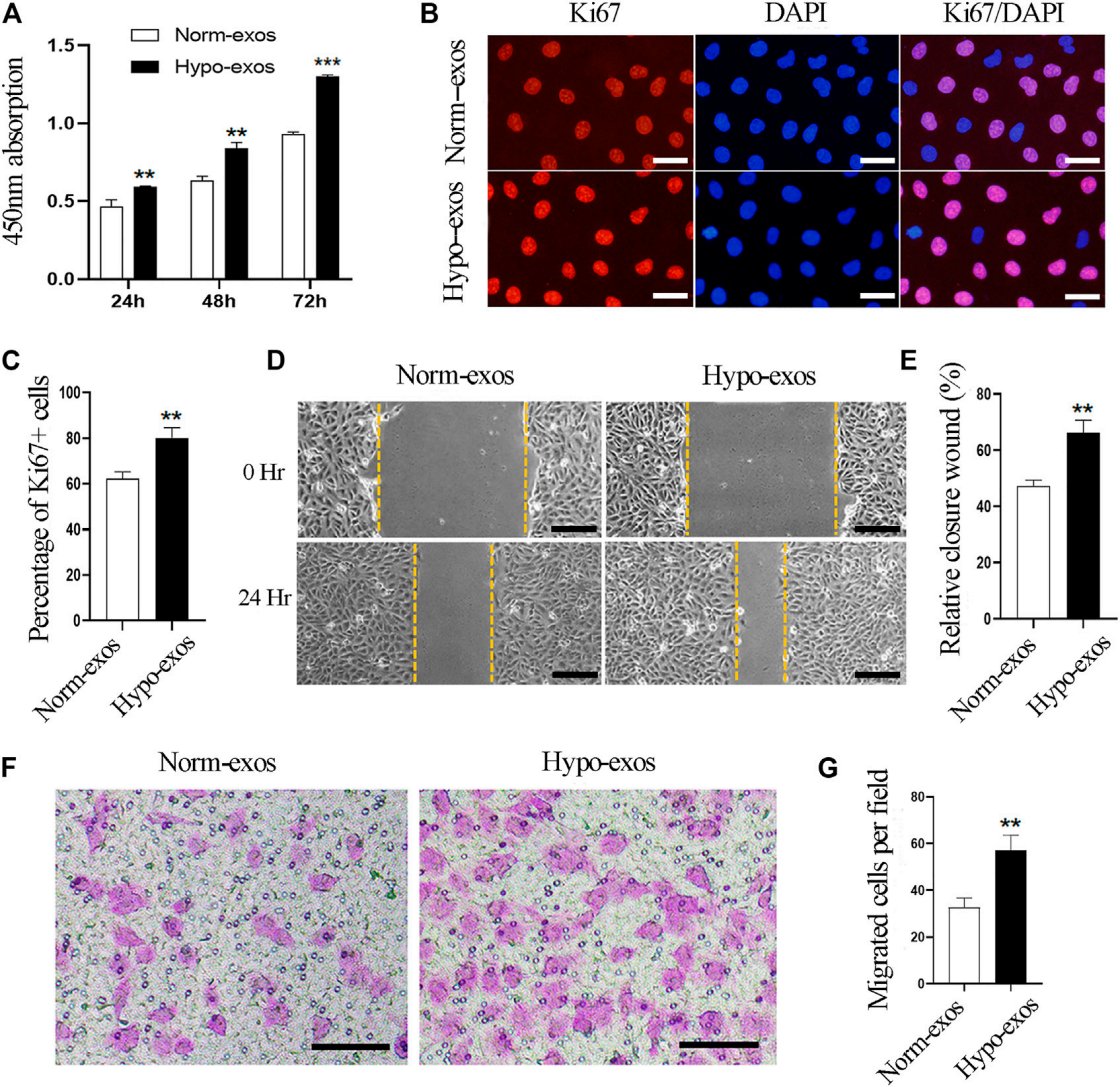
Hypo-exos significantly enhance the growth and migration of endothelial cells *in vitro* compared with Norm-exos. **(A)** Effects of Hypo-exos and Norm-exos on the viability of endothelial cells analyzed using the CCK-8 assay. **(B)** Effects of Hypo-exos and Norm-exos on the proliferation of endothelial cells assessed by immunofluorescence staining of Ki67; scale bars = 100 μm. **(C)** Percentage of Ki67-positive cells (%) quantified in **(B)**. **(D)** Significantly increased closure of the scratched area in Hypo-exos treated group was observed in wound healing assays; scale bars = 200 μm. **(E)** Quantification of the rate of scratch closure (%) in **(D)**. **(F)** Significantly higher number of endothelial cells in Hypo-exos group migrated to the bottom than Norm-exos group verified by Transwell migration assays; scale bars = 200 μm. **(G)** Quantification of the number of migrated HUVECs in **(F)**. **p < 0.01, ***p < 0.001.

To explore the biologic functions of SHED-derived exosomes, we used CCK8 assays to evaluate the viability of HUVEC cells after incubation with Hypo-exos or with Norm-exos. The results showed that treatment with Hypo-exos significantly enhanced the growth of endothelial cells, and the positive effect became more significant at 48 and at 72 h compared with Norm-exos (Figure 2A). Next, in order to examine whether Hypo-exos have an increased influence on cell proliferation, immunofluorescent staining for Ki67 was carried out, which revealed that the rate of Ki67-positive cells was 80.2 ± 4.4% in the Hypo-exos treated cells, which was significantly higher than that of the Norm-exos treated cells (62.1 ± 3.0%) (Figures 2B,C). In sum, these data suggested that Hypo-exos significantly enhance endothelial cell growth *in vitro* compared with Norm-exos.

Additionally, it has been demonstrated that the migration and invasion of endothelial cells are necessary processes involved in angiogenesis (Mostmans et al., 2017). Therefore, we next examined whether Hypo-exos could improve the migratory behavior of HUVECs *in vitro* compared with Norm-exos. In order to do this, we performed scratch wound healing assays and observed that endothelial cells treated with Hypo-exos had a significantly higher rate of wound closure than Norm-exos treated cells (Figures 2D,E). To further verify this observation, we performed transwell migration assays, which showed that a significantly higher number of endothelial cells in the Hypo-exos treated group migrated to the bottom compartment compared to the treatment with Norm-exos (Figures 2F,G). These findings indicate that Hypo-exos can enhance the migration and invasion of endothelial cells *in vitro* compared with Norm-exos.

Overall, these data demonstrate that Hypo-exos can significantly enhance the growth and migration of endothelial cells *in vitro* compared with Norm-exos.

### Hypo-Exos Significantly Promote the Tube Formation of Endothelial Cells *In Vitro* Compared With Norm-Exos

**FIGURE 3:**
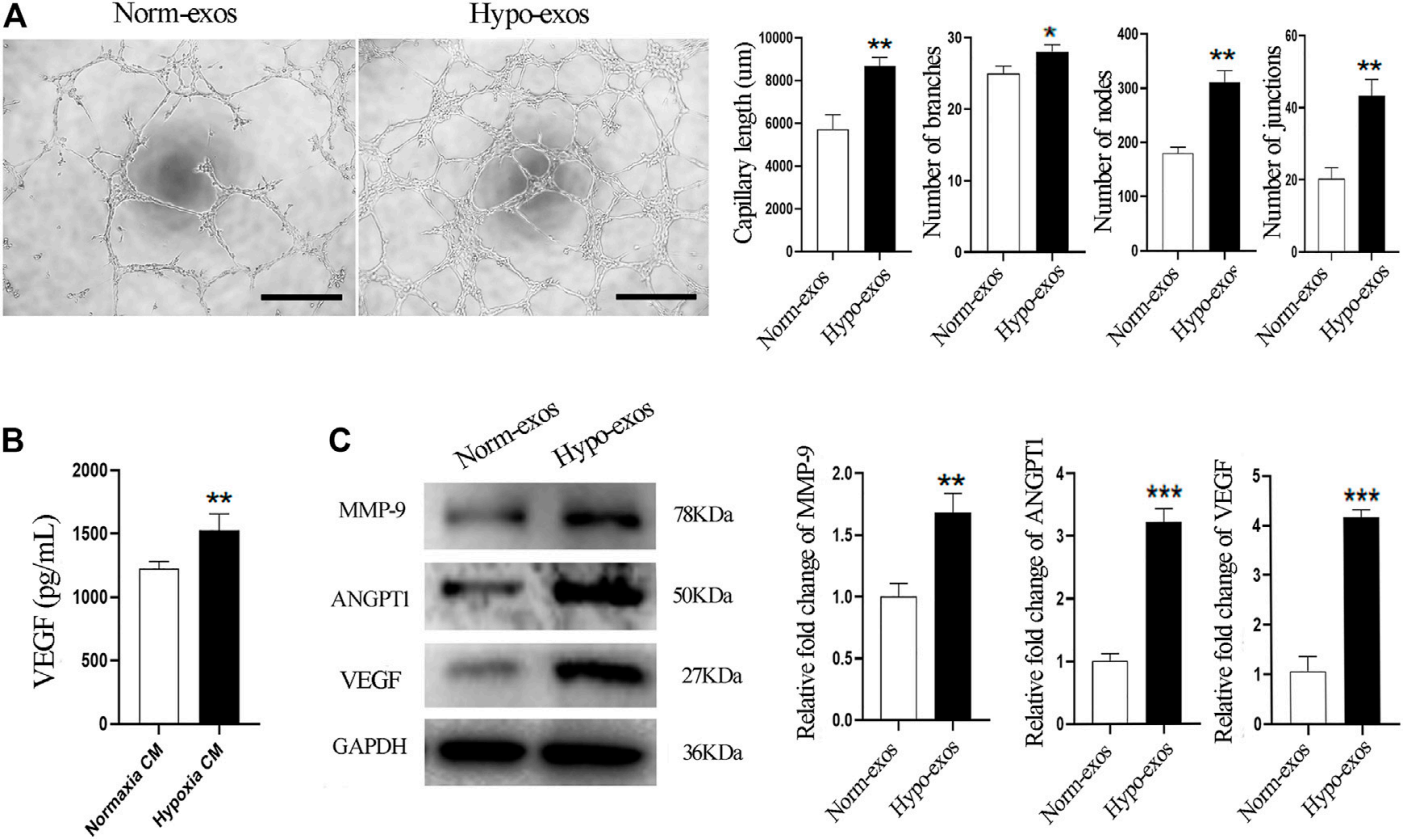
Hypo-exos significantly enhance the tube formation of endothelial cells *in vitro*. **(A)** Significantly enhanced effect in the Hypo-exos treated group on the formation of network structures by HUVECs shown by tube formation assays (left) and the formed capillary-like structures in two groups were quantitated (right); scale bars = 500 μm. **(B)** Secretory VEGF levels at 48 h were increased significantly in hypoxic conditioned medium as detected by ELISA. **(C)** Protein expression levels of MMP-9, ANGPT1 and VEGF were enhanced markedly as shown by western blot analysis (left) and relative levels were qualified as relative fold change to the respective Norm-exos treated cells (right). *p < 0.05, **p < 0.01, ***p < 0.001.

Based on the previous observations of the positive effect of Hypo-exos on the growth and migration of endothelial cells, two important processes involved in angiogenesis (Mostmans et al., 2017), we explored the potential of Hypo-exos to promote the angiogenesis ability of endothelial cells. We performed tube formation assays *in vitro,* which revealed a better effect on the tube formation of HUVECs in the Hypo-exos treated group. Significantly longer tubes and many more branches, nodes and junctions of the network structures formed by endothelial cells were observed in the Hypo-exos treated cells compared with the Norm-exos treated cells (Figure 3A). Next, we compared the protein concentration of VEGF, which is a crucial factor in angiogenesis (Scott et al., 2015), in the conditioned medium collected from SHED cells cultured under hypoxic or normoxic conditions. The quantitative ELISA results showed that hypoxia treatment resulted in a significant increase of VEGF secretion at a concentration of 1525.0 ± 130.2 pg/ml compared with that of the normoxic culture (1221.9 ± 59.4 pg/ml) (Figure 3B). Subsequently, we treated HUVECs with Hypo-exos or with Norm-exos for 36 h and examined the expression level of angiogenesis-related factors. Western blot analysis showed that proangiogenic factors, including VEGF, MMP-9 and ANGPT1, were significantly upregulated by treatment with Hypo-exos (Figure 3C). Taken together, these results demonstrate that Hypo-exos enhance the tube formation of endothelial cells *in vitro* associated with the upregulation of proangiogenic factors.

### Hypo-Exos Significantly Enhance Micro-Vessel Formation *In Vivo*

**FIGURE 4:**
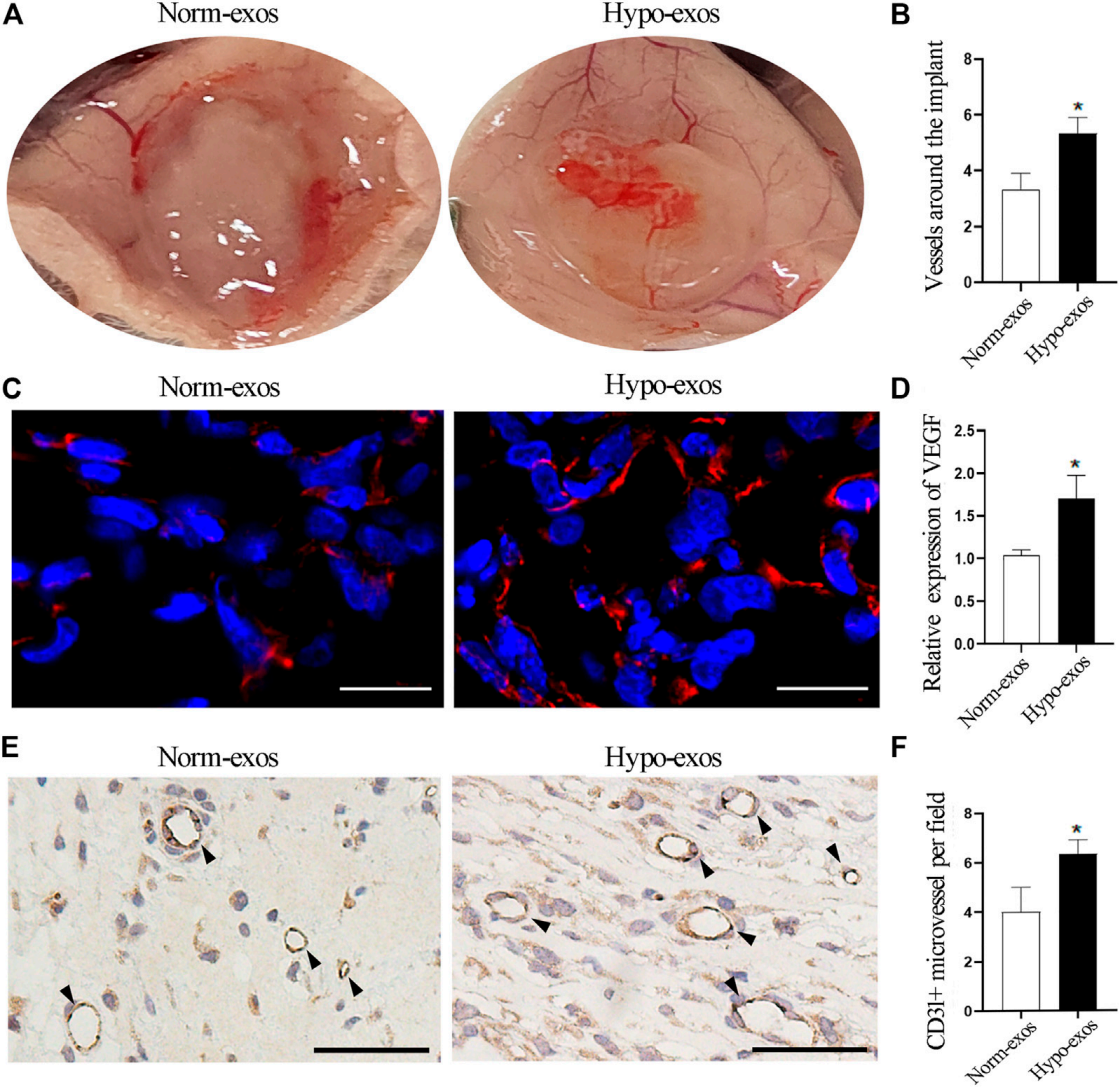
Hypo-exos promote micro-vessel formation *in vivo* compared with Norm-exos. **(A)** Representative images of Matrigel plugs implanted subcutaneously in mice for 10 days. **(B)** Quantification of the number of micro-vessels around the plug implants. **(C)** Immunofluorescence staining for VEGF in implanted matrigel plugs containing Hypo-exos or Norm-exos. Scale bars = 500 μm. **(D)** Quantification of VEGF expression in Matrigel plugs. **(E)** Increased neovasculature in matrigel plugs containing Hypo-exos visualized by immunofluorescence staining of CD31; scale bars = 500 μm. **(F)** Quantification of the number of tubular structures stained by CD31. *p < 0.05.

Next, to evaluate whether Hypo-exos also have a significantly enhanced effect on micro-vessel formation *in vivo*, we performed matrigel plug assays, a classic murine model to examine the angiogenesis ability of endothelial cells (Zeng et al., 2019). Five hundred μl matrigel containing 100 μg Hypo-exos or Norm-exos were mixed with 1 × 10^7^ HUVECs and then were subcutaneously injected into the dorsal skin of mice. At 10 days after injection, the mice were sacrificed, and the number of micro-vessels formed around the matrigel plugs was calculated. The results revealed that the number of micro-vessels around the matrigel plugs was significantly increased in the Hypo-exos treated group (Figures 4A,B). After removing the matrigel plugs, a redder appearance was observed in plugs containing Hypo-exos (100 μg) than in plugs containing Norm-exos (Supplementary Figure S2). To further visualize the increased neovasculature in the matrigel containing Hypo-exos, immunofluorescence staining for VEGF and immunohistochemical staining for CD31 were performed. The results showed that a higher expression of VEGF and a higher number lumenal structures stained by CD31 were displayed in the Hypo-exos group (Figures 4C–F). Taken together, the results demonstrated that treatment with Hypo-exos significantly enhanced micro-vessel formation *in vivo* compared to treatment with Norm-exos.

### let-7f-5p and miR-210-3p are Transferred by Hypo-Exos

**FIGURE 5:**
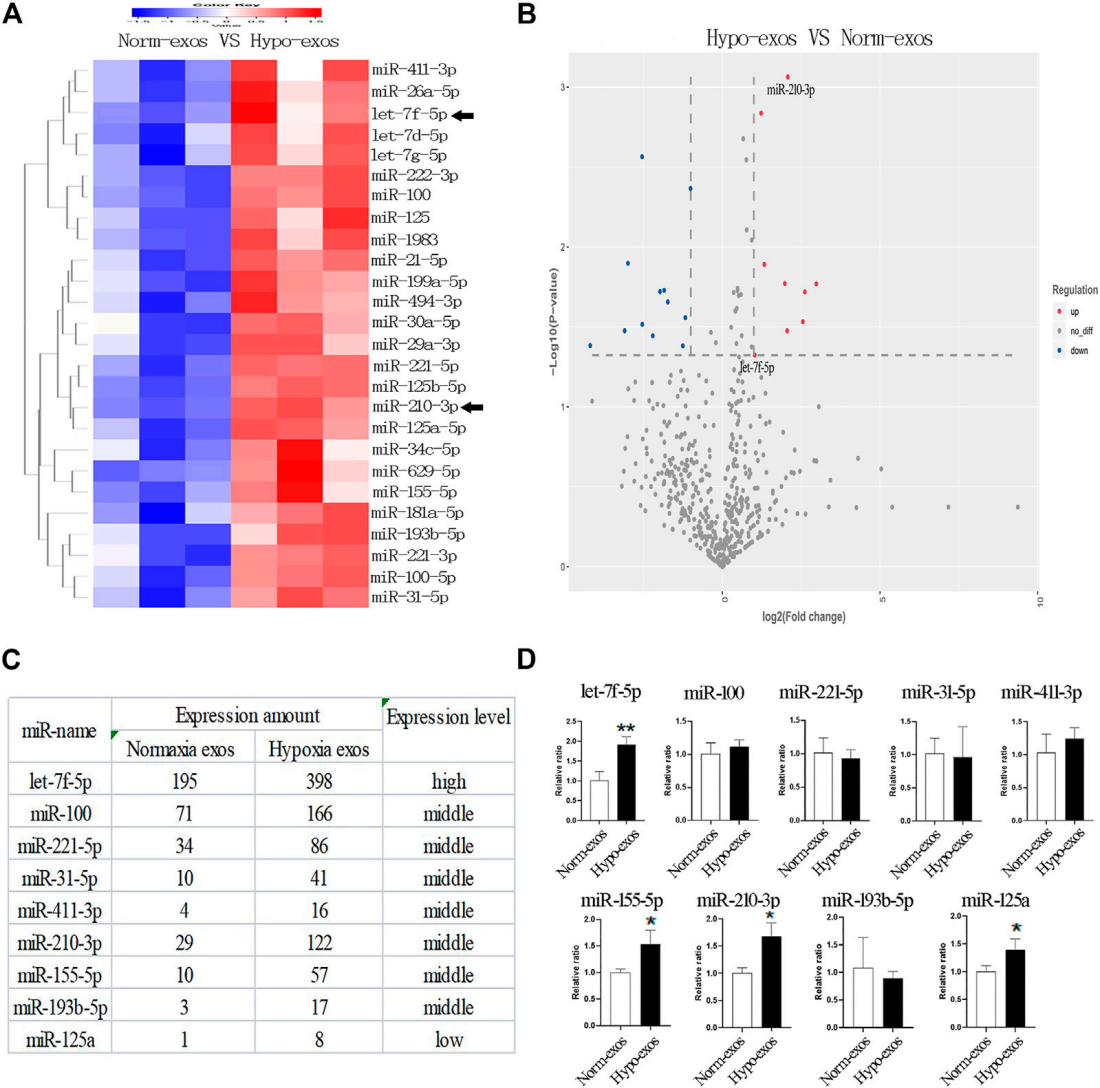
Profiling and identification of upregulated miRNAs in Hypo-exos and in Norm-exos. **(A)** Heat map showing the profiles of upregulated miRNAs in Hypo-exos compared with Norm-exos. **(B)** Volcano map revealing nine significantly upregulated miRNAs with a ≥2 fold difference between Hypo-exos and Norm-exos. **(C)** The nine significantly upregulated miRNAs (≥2 fold, *p* < 0.05) are listed. **(D)** Comparison of let-7f-5p, miR-100, miR-221-5p, miR-31-5p, miR-411-3p, miR-210-3p, miR-155-5p, miR-193b-5p and miR-125a between Norm-exos and Hypo-exos by qRT-PCR. *p < 0.05, **p < 0.01.

To understand the potential molecular mechanism responsible for the positive effects of Hypo-exos, we performed exosomal miRNA sequencing and compared the expression profiles of miRNAs between Hypo-exos and Norm-exos. Because exosomes encapsulate donor cell-derived bioactive factors that mediate intercellular communications, we primarily focused on the upregulated miRNAs in Hypo-exos compared with Norm-exos. Heat map analysis revealed that there were 26 significantly upregulated miRNAs in Hypo-exos compared with Norm-exos (*p* < 0.05) (Figure 5A). According to the miRNA sequencing analysis, we concentrated on nine significantly upregulated miRNAs with a ≥2 fold difference between Hypo-exos and Norm-exos, which included let-7f-5p, miR-100, miR-221-5p, miR-31-5p, miR-411-3p, miR-210-3p, miR-155-5p, miR-193b-5p, and miR-125a (Figures 5B,C). We then validated the expression levels of those nine miRNAs and found that four of them, let-7f-5p, miR-155-5p, miR-210-3p, and miR-125a, were significantly upregulated in Hypo-exos (Figure 5D). We next searched the literature and focused on let-7f-5p and miR-210-3p, which have reported in previous studies to be involved in angiogenesis (Wang et al., 2020; Zhu et al., 2020), and importantly, both of those miRNAs have been shown to be related to hypoxia treatment (Zhang et al., 2009; Lu et al., 2019; Chen et al., 2020).

### let-7f-5p and miR-210-3p Enhance the Tube Formation Ability of Endothelial Cells

**FIGURE 6:**
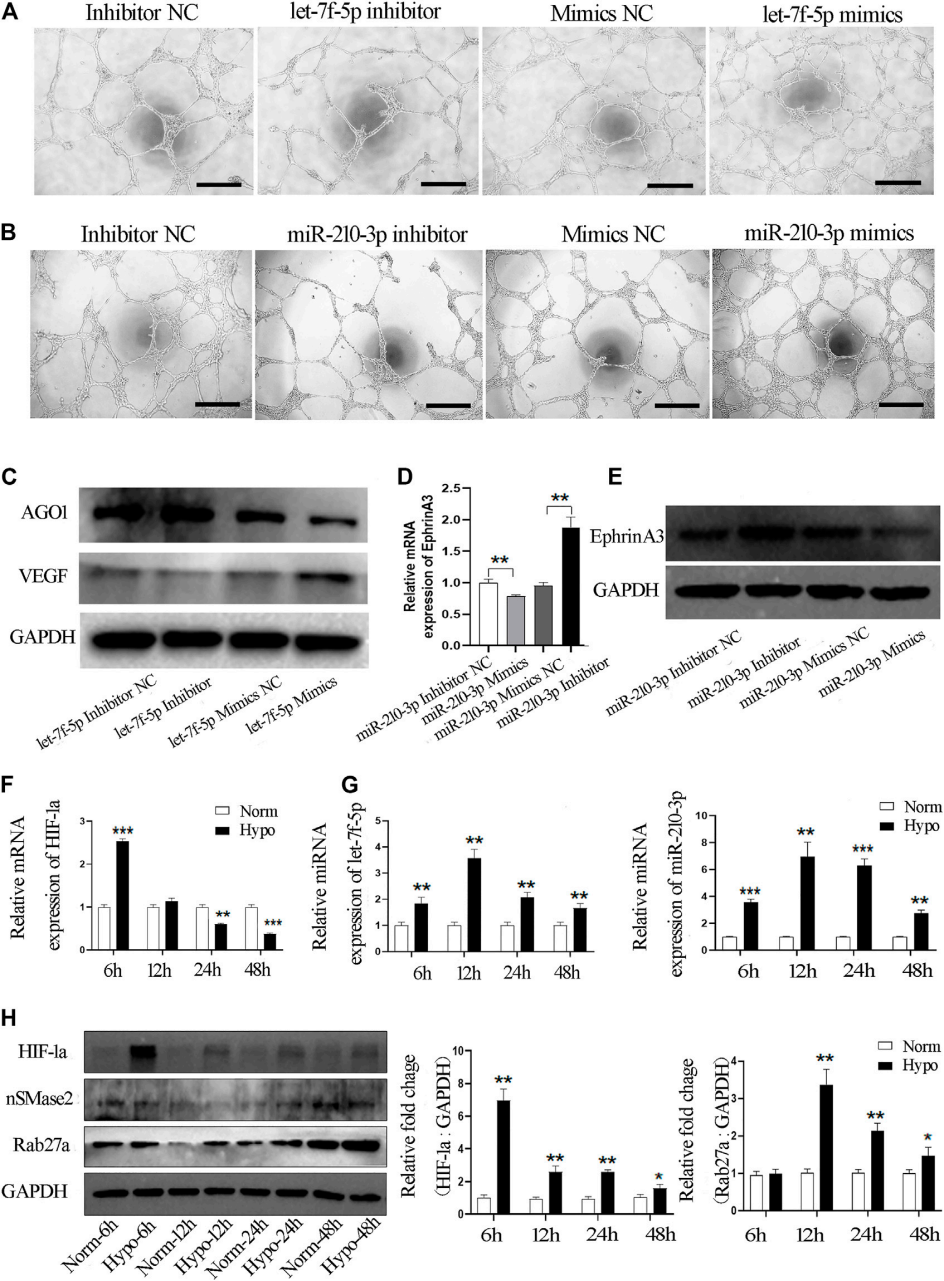
Hypo-exos transfer both let-7f-5p and miR-210-3p to promote the tube formation of endothelial cells. **(A)** Matrigel tube formation assay of HUVECs transfected by mimics, inhibitor and NC of let-7f-5p; scale bars = 500 μm. **(B)** Matrigel tube formation assay of HUVECs transfected by mimics, inhibitor and NC of miR-210-3p; scale bars = 500 μm. **(C)** Expression of AGO1 and VEGF validated in endothelial cells transfected by mimics, mimics NC, inhibitor and inhibitor NC of let-7f-5p by qRT-PCR. **(D)** Expression of EphrinA3 validated in endothelial cells transfected by mimics, mimics NC, inhibitor and inhibitor NC of miR-210-3p by qRT-PC. **(E)** Expression of EphrinA3 was verified in HUVECs transfected with mimics, mimics NC, inhibitor and inhibitor NC of miR-210-3p by Western blot. **(F)** Expression of HIF-1a and **(G)** let-7f-5p and miR-210-3p in SHED cells exposed to hypoxic treatment at 6, 12, 24, and 48 h verified by qRT-PCR. **(H)** Expression of HIF-1a, nSMase 2 and Rab27a in SHED cells exposed to hypoxic treatment at 6, 12, 24, and 48 h verified by Western blot (left). Quantification of the relative levels of HIF-1a and Rab27a as relative fold change to the respective control cells (right). *p < 0.05, **p < 0.01, ***p < 0.001.

Next, we evaluated whether let-7f-5p and/or miR-210-3p alter the ability of vascular tube formation by endothelial cells *in vivo* by transfecting miRNA specific mimics, inhibitors or negative control into HUVECs. We observed that significantly shorter capillary length and fewer branches, nodes and junctions of vascular tubes were formed by endothelial cells transfected with the let-7f-5p inhibitor and an obviously promoting effect on the vascular tube formation was seen in the mimics treated group (Figure 6A, Supplementary Figure S3). Similar results were observed when the endothelial cells were transfected with the miR-210-3p inhibitor or the mimics (Figure 6B, Supplementary Figure S4). In sum, these data demonstrated that both let-7f-5p and miR-210-3p can regulate the tube formation ability of endothelial cells.

We then examined how let-7f-5p enhances the angiogenesis of endothelial cells. It has been reported that hypoxia-induced miRNAs target argonaute 1 (AGO1) to promote angiogenesis (Chen et al., 2013). VEGF is a well-established factor with pro-angiogenic activity and AGO1 has been reported to regulate VEGF mRNA transcription and protein synthesis (Eswarappa et al., 2014). In order to verify the involvement of that pathway, we transfected the inhibitor or mimics of let-7f-5p into endothelial cells and western blot analysis showed that the expression of AGO1 was significantly increased and the level of VEGF was obviously reduced in endothelial cells transfected with the let-7f-5p inhibitor, and opposite results were found in the let-7f-5p mimics-treated cells (Figure 6C). Therefore, these results confirmed that let-7f-5p can enhance angiogenesis *via* the AGO1/VEGF signal pathway.

Subsequently, as shown at http://microRNA.org, EphrinA3 is a direct target of miR-210-3p, and this finding has been validated by many previous studies (Fasanaro et al., 2009; Wang N et al., 2017; Wang et al., 2020). Further, it has also been proven that ephrinA3 can directly affect the angiogenic ability of endothelial cells (Hu et al., 2010; Ujigo et al., 2014). Based on those studies, we examined the expression level of ephrinA3 in HUVECs using qRT-PCR and western blot analyses. miR-210-3p inhibition significantly increased the mRNA and protein expression level of ephrinA3, whereas overexpression of miR-210-3p dramatically downregulated the expression of ephrinA3 (Figures 6D,E). Taken together, these results indicated that miR-210-3p enhances the angiogenesis ability of endothelial cells, likely through the miR-210-3p/ephrinA3 signaling pathway.

Moreover, it is well-known that the let-7 family and miR-210-3p are hypoxia-responsive microRNAs (Zhang et al., 2009; Chen et al., 2020). HIF-1a is a crucial factor that mediates the regulation of hypoxia-inducible genes (Majmundar et al., 2010). We examined the mRNA expression level of HIF-1a in SHED cells exposed to hypoxia and found that the expression of HIF-1a mRNA was significantly increased at 6 h (Figure 6F). We validated the expression of let-7f-5p and miR-210-3p in SHED cells exposed to hypoxia at different time points and observed that the hypoxia treatment increased the expression of let-7f-5p and miR-210-3p in SHED cells, especially at 12 h (Figure 6G). Finally, we explored why the hypoxia treatment increased the release of exosomes. It has been reported that Rab GTPase family member Rab27a and nSMase2 are key mediators of the biogenesis and secretion of exosomes (Sahoo and Losordo, 2014). Western blot analysis showed that the protein expression level of Rab27a was significantly increased in hypoxia-treated SHED cells compared with normoxia-treated SHED cells (Figure 6H).

## Discussion

With the recent development of technologies, MSCs have been shown to have an increasingly beneficial potential in regenerative medicine and immunomodulation. When MSCs are delivered *in vivo*, it was observed that the majority of intravenously administered MSCs are trapped in capillaries of the liver or lung and only a relatively few MSCs reached the injury target sites (Kraitchman et al., 2005; François et al., 2006; Toma et al., 2009). Other risks of MSC transplants have also been reported including graft rejection, induction of tumor growth and granulocytosis (Tögel et al., 2004; Jeong et al., 2011; Fennema et al., 2018). Hence, many disadvantages remain to be overcome before MSC transplantation can be widely used clinically.

It has been pointed out that paracrine mechanisms play important roles in the therapeutic functions of MSC transplantation (Mathieu et al., 2019). MSC-derived exosomes can transport diverse signaling factors to recipient cells to regulate cell proliferation and/or angiogenesis and overcome the disadvantages of direct MSC transplantation (Balaj et al., 2011; Webber et al., 2015; Qin et al., 2016). It is known that SHED cells are in relatively hypoxic conditions during the recession of dental pulp tissue in deciduous teeth with physiological root resorption. Therefore, we mimicked those physiological conditions by hypoxia preconditioning (2% O_2_) for cell culture in our study to explore the underlying mechanism of hypoxic SHED cell-derived exosomes on angiogenesis.

The present study revealed a similar morphology between Hypo-exos and Norm-exos. However, nanoparticle tracking analysis revealed that Hypo-exos are significantly larger than Norm-exos, consistent with the finding in hypoxic preconditioned bone MSCs (Liu et al., 2020b). Besides, it was also demonstrated that Hypo-exos can enhance the proliferation and migration activities of endothelial cells, two important biological activities involved in angiogenesis. Additionally, Hypo-exos significantly promoted the tube formation of endothelial cells *in vitro* and levels of proangiogenic factors, including VEGF, MMP-9 and ANGPT1, were significantly upregulated. VEGF has been reported to contribute to the transition of endothelial cells from the quiescent to the activated state at the onset of the sprouting process in angiogenesis (Watson et al., 2017). The significantly positive effects on angiogenesis of Hypo-exos compared with Norm-exos were also verified by matrigel plug assays *in vivo* (Zeng et al., 2019).

We next explored the underlying mechanism responsible for these positive effects of Hypo-exos. miRNAs are important contents of exosomes and can regulate protein synthesis by binding to the 3′-untranslated regions of target mRNAs (Didiano and Hobert, 2008). Therefore, we performed exosomal miRNA sequencing and compared the expression profiles of miRNAs between the two types of exosomes. Our results validated that let-7f-5p and miR-210-3p are abundant in Hypo-exos.

It has been reported that HIF-1a is an important transcription factor that regulates hypoxic responses, and which is necessary for the expression of specific miRNAs under hypoxic culture, for example, miR-23a and miR-135b (Umezu et al., 2014; Li et al., 2019). As a type of hypoxic-related microRNA, let-7f has been demonstrated to play diverse roles in regulating cell growth, invasion, migration and angiogenesis in tumors (Li et al., 2016; Gao et al., 2018). In our study, we targeted let-7f-5p, which is a member of the let-7 family. We demonstrated that let-7f-5p regulates angiogenesis of endothelial cells *via* the AGO1/VEGF signaling pathway, which is consistent with a previous finding that the let-7 family, which is enriched in hypoxic human adipose tissue-derived MSCs, contributes to angiogenesis (Zhu et al., 2020). The different subtypes of let-7 family members may have different biological functions and the exact subtype of let-7 family contribution to angiogenesis needs further study.

Of relevance to our finding, miR-210 has been reported to be increased by hypoxic exposure (Wang H et al., 2014). Additionally, it was also revealed that oral squamous cell carcinoma-derived exosomes upregulate miR-210-3p targeting ephrinA3 to enhance oral cancer angiogenesis through the PI3K/AKT signaling pathway (Wang et al., 2020). Consistent with this, we observed that the increased expression of miR-210-3p decreases ephrinA3 expression in HUVECs and enhances tube formation. Our results are in accordance with several reports suggesting that miR-210 produces pro-angiogenesis effects *via* an ephrinA3 dependent mechanism (Wang L et al., 2017) or the HIF/VEGF/Notch signaling pathway (Liang et al., 2020). It’s worth noting that ephrinA3 can also affect the angiogenic ability of endothelial cells to influence angiogenesis (Hu et al., 2010; Ujigo et al., 2014).

Hypoxia preconditioning increases exosome release (King et al., 2012). However, how hypoxia regulates exosome release has not been fully clarified. Sphingolipid-metabolizing enzymes, such as the Rab GTPase family Rab27a/b and nSMase2, are key mediators of the biogenesis and secretion of exosomes (Ostrowski et al., 2010; Kowal et al., 2014). We found that the protein expression level of Rab27a was significantly increased in hypoxia-treated SHED cells compared with normoxic-treated SHED cells. Similarly, hypoxia-preconditioned extracellular vesicles derived from breast cancer cells were shown to be regulated by the HIF-dependent expression of the small GTPase Rab22a (Wang T et al., 2014). The mechanism of hypoxia-regulated exosomes release need to be further studied.

## Data Availability

The original contributions presented in the study are included in the article/Supplementary Material, further inquiries can be directed to the corresponding authors.
