# Supplementary material for: Exosomes Derived From Hypoxia-Conditioned Stem Cells of Human Deciduous Exfoliated Teeth Enhance Angiogenesis via the Transfer of let-7f-5p and miR-210-3p
Source: Front Cell Dev Biol. 2022 Apr 26;10:879877. doi: 10.3389/fcell.2022.879877 (PMC9086315; doi:10.3389/fcell.2022.879877)
Supplement: Supplementary file 1 [file DataSheet1.docx]

**SUPPLEMENTAL FIGURES, FIGURE LEGENDS AND SUPPLEMENTAL TABLES**


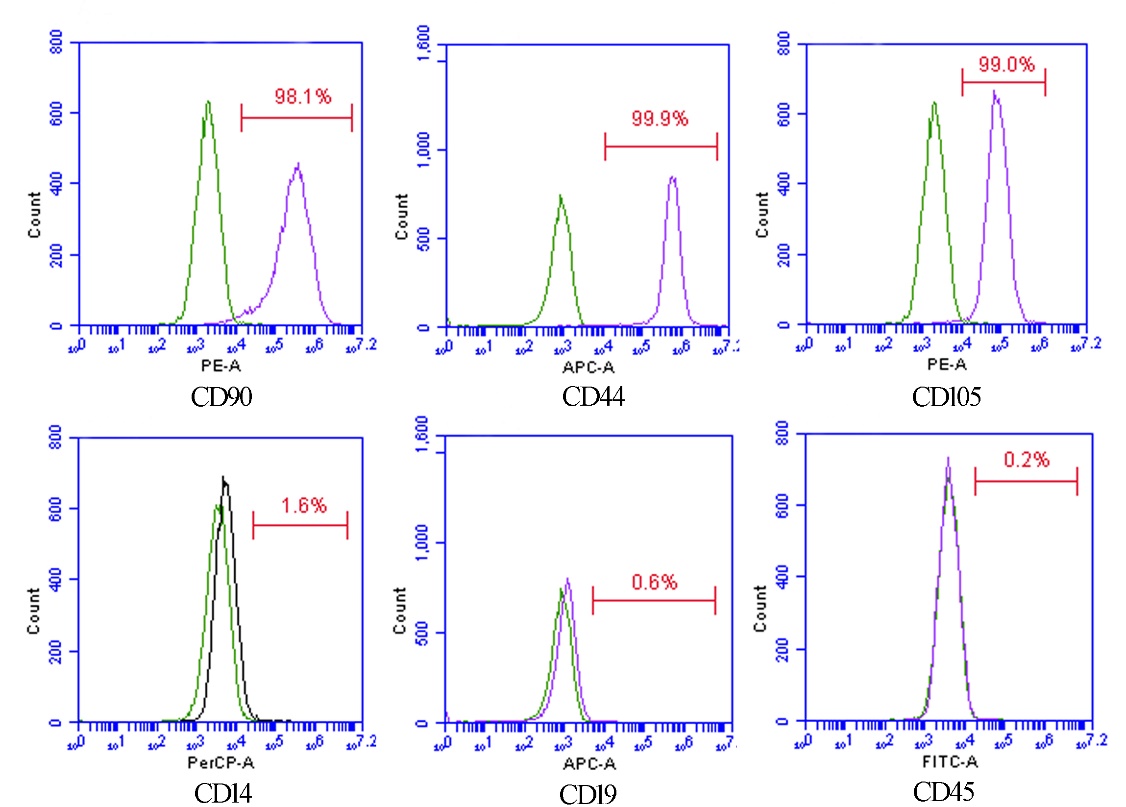


**Supplementary Figure 1** Surface markers of SHED cells analyzed by flow cytometry were positive for mesenchymal markers (CD44, CD105 and CD90) but negative for endothelial markers (CD45, CD19 and CD14).

**
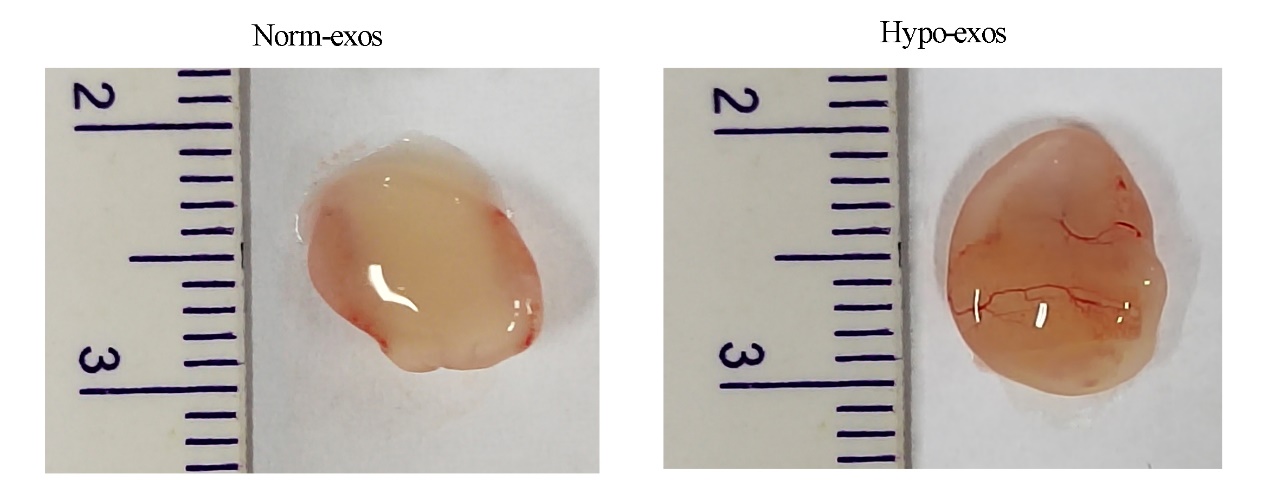
**

**Supplementary Figure 2** Representative images of Matrigel plugs. A redder appearance was observed in Matrigel plugs containing Hypo-exos than in those with Norm-exos.


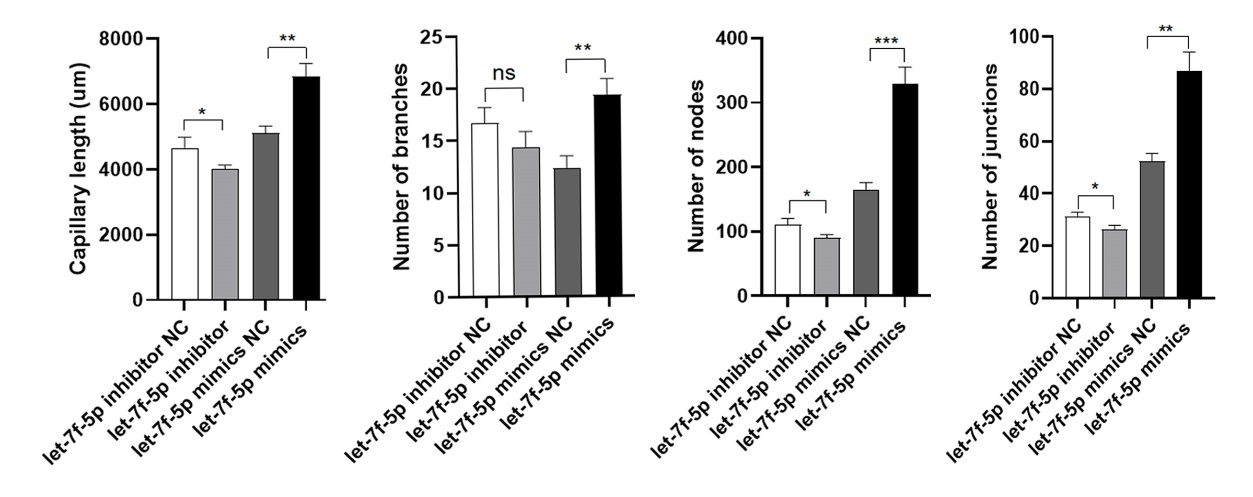


**Supplementary Figure 3** Hypo-exos transfer let-7f-5p to promote the tube formation of endothelial cells. Quantification of capillary length, branches, tube nodes and functions in the network structures of HUVECs shown in FIGURE 6A. P values are indicated with “*”, *P < 0.05, **P < 0.01, ***P < 0.001.

1


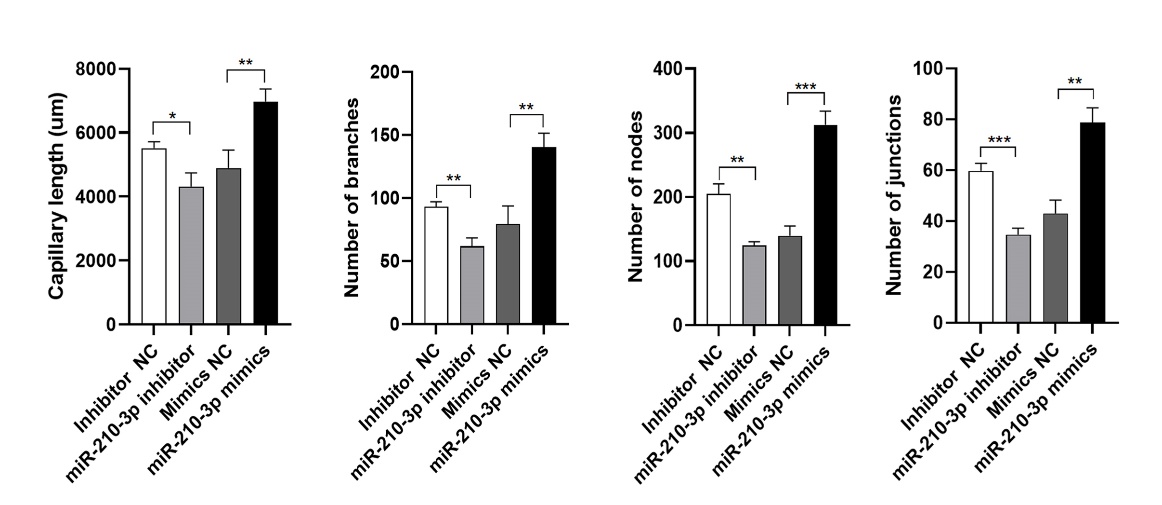


**Supplementary Figure 4** Hypo-exos transfer miR-210-3p to promote the tube formation of endothelial cells. Quantification of capillary length, branches, tube nodes and functions in the network structures of HUVECs shown in FIGURE 6B. P values are indicated with “*”, *P < 0.05, **P < 0.01, ***P < 0.001.

**Supplementary Table 1** Oligo sequences used for mRNA qRT-PCR analysis

| Gene Name | Forward primer | Reverse primer |
| --- | --- | --- |
| GAPDH | ATGACTCTACCCACGGCAAG | GGAAGATGGTGATGGGTTTC |
| HIF-1a | CCGCTGGAGACACAATCATA | GGTGAGGGGAGCATTACATC |
| EphrinA3 | TGCAACGCACAGACACTTT | ATAGGGTGAGCAGGGCAA |
